# Daytime sleepiness in Parkinson's disease: a multifaceted symptom

**DOI:** 10.3389/frsle.2023.1302021

**Published:** 2023-12-22

**Authors:** Felice Di Laudo, Luca Baldelli, Greta Mainieri, Giuseppe Loddo, Angelica Montini, Caterina Pazzaglia, Monica Sala, Francesco Mignani, Federica Provini

**Affiliations:** ^1^Department of Biomedical and NeuroMotor Sciences (DiBiNeM), University of Bologna, Bologna, Italy; ^2^IRCCS Istituto delle Scienze Neurologiche di Bologna, Bologna, Italy; ^3^Department of Primary Care, Azienda USL di Bologna, Bologna, Italy

**Keywords:** excessive daytime sleepiness (EDS), sleep questionnaire, Parkinson's disease, video polysomnography, sleep hygiene, dopamine agonist

## Abstract

Excessive daytime sleepiness is a symptom experienced by more than one-third of patients with Parkinson's disease and is associated with disease duration and severity, dopaminergic therapy, and several non-motor symptoms. In recent years, growing evidence has been suggesting “primary” sleepiness as a symptom in Parkinson's disease due to common pathophysiological features: for this reason, it is crucial to recognize sleepiness in these patients and to investigate and exclude other conditions possibly leading to sleepiness (e.g., heavy dopaminergic therapy or breathing disorders during sleep). For both inpatients and outpatients, the key to a correct diagnosis is a structured clinical interview, together with questionnaires, for a better characterization of symptoms and the use of objective measures as the most precise method to assess excessive daytime sleepiness. Finally, there are some therapeutical approaches that may be attempted for these patients, and although there is still no consensus on a standardized therapy, clinical trials with new drugs are currently persevered on.

## Introduction

Sleepiness is a physiological state experienced by most individuals over a 24-h period. However, sleepiness occurring at inappropriate times, with increased frequency or interfering with daily functioning is usually considered excessive (Martin et al., 2023). According to the last version of the International Classification of Sleep Disorders (ICSD-3-TR) (American Academy of Sleep Medicine, 2023), excessive daytime sleepiness (EDS) is defined as the inability to maintain wakefulness and alertness during the major waking episodes of the day, with sleep occurring unintentionally or at inappropriate times almost daily for at least 3 months. Sleepiness can be defined objectively as “sleep propensity,” i.e., the tendency to fall asleep; however, no consensus exists regarding the precise threshold above which it becomes excessive. EDS appears in a heterogeneous group of primary or secondary disorders of hypersomnolence. Secondary EDS may appear in both neurological and non-neurological (immunological, paraneoplastic, and vascular) disorders. Among neurological disorders, EDS is present in many neurodegenerative diseases, in particular Parkinson's disease (PD). EDS represents one of the most relevant non-motor symptoms affecting these patients, with evidence suggesting that EDS might also precede PD diagnosis (Leng et al., 2018). Moreover, the importance of recognizing PD phenotypes with prominent daytime sleepiness lies not only in the possibility of tailoring PD treatment but also in the exclusion of concomitant sleep disorders that might lead to EDS. However, EDS in PD is not only secondary to sleep disorders or PD medications, but growing evidence also suggests a primary etiology within PD itself. Indeed, Sauerbier et al. (2016) proposed EDS as a prominent feature of one specific PD subtype, the so-called Park-sleep subtype, characterized by clinically impacting EDS, a prominent susceptibility to dopamine agonists' sedative effect, and being at particular risk of sudden onset of sleep during therapy (Tall et al., 2023).

Within this framework, this narrative review aimed to define the impact of EDS in PD in terms of epidemiology and pathophysiological characteristics, to propose a diagnostic algorithm, and to illustrate current therapeutical approaches.

## Epidemiology

### Prevalence

The prevalence of EDS in patients with PD varies from 10 to 70%. A recent meta-analysis (comparing 40 studies with a total of 6,673 patients) found a pooled EDS rate of 35% [95% CIs (30%–40%)]. The wide range is due to the different evaluation methods used: EDS has been identified subjectively with questionnaires or objectively by using polysomnography (PSG). Subjective questionnaires have shown a pooled prevalence of EDS in PD patients of 33% among different studies (Maggi et al., 2023). Both the Epworth Sleepiness Scale (ESS, with a proposed cutoff of >10 to identify pathologic daytime sleepiness (Johns, 1994) and the SCale for Outcomes in PArkinson's disease (SCOPA)-SLEEP daytime sleepiness (DS) subscale (with a cutoff of >4; Marinus et al., 2003) have been validated for screening and measuring the severity of daytime sleepiness in PD patients (Högl et al., 2010). Finally, item 15 in the PD Sleep Scale (PDSS; Chaudhuri et al., 2002; Martinez-Martin et al., 2008), pertaining to daytime sleepiness, has shown a correlation with SCOPA-SLEEP-DS and abnormal sleep patterns in overnight polysomnography (Dhawan et al., 2006); thus, PDSS has been proposed as a screening tool for daytime sleepiness in PD patients (Kurtis et al., 2018).

It may be useful to underline the fact that fatigue may mimic daytime sleepiness due to possible ambiguity in terminology used by patients: a general lack of energy during the day, often related to muscular weakness, may be reported as “sleepiness,” and it is crucial for clinicians to further investigate the symptom.

Objective polysomnographic methods have shown a pooled prevalence of EDS in PD patients of 44% (Maggi et al., 2023). The most used are the Multiple Sleep Latency Test (MSLT; Littner et al., 2005) for measuring the amount of time the patient needs to fall asleep and the Maintenance of Wakefulness Test (MWT; Mitler et al., 1982) for assessing the ability to remain awake under soporific conditions.

Some authors have suggested that combining subjective and objective measures (Bargiotas et al., 2019) could be a more precise way to diagnose EDS in PD patients, because the common comorbidity of cognitive impairment (Baiano et al., 2020) or fatigue (Valko et al., 2010) may underestimate or overestimate, respectively, the presence of EDS. Nevertheless, some studies have used MSLT and ESS together and have shown differences in identifying daytime sleepiness, with higher rates of EDS if assessed with the ESS (Cochen De Cock et al., 2014) or no significant correlation between the results using the two methods (Bargiotas et al., 2019) In contrast, a recent meta-analysis (Maggi et al., 2023) has shown a higher pooled prevalence of EDS when assessed with polysomnography than subjective questionnaires, pointing out again that the prevalence could differ among studies based on whether objective or subjective methods are used to identify EDS, as they explore different aspects of sleepiness.

### Risk factors

As disentangling the different causes of EDS is necessary to manage this symptom appropriately (Marinus et al., 2018), it is important to underline that male sex, longer PD disease duration and severity, higher levodopa equivalent daily dose (LEDD), and the presence of autonomic and depressive symptoms increase the risk of EDS in patients with PD. Regarding sex differences, some studies have suggested that male PD patients appeared to experience EDS more than female PD patients (Feng et al., 2021; Liu et al., 2021). However, a higher risk of EDS was found in the general population in men aged more than 60 years compared to men younger than 60 years. Therefore, this association seems to not be specific to PD. The relationship between aging and EDS in PD patients is controversial, with some studies reporting more EDS in older PD patients (Zhu et al., 2016) but not others (Amara et al., 2017).

Longer disease duration is associated with EDS in PD patients, as demonstrated by a longitudinal study showing that 46% of PD patients without EDS at baseline developed EDS after a 5-year follow-up (Zhu et al., 2016) and another study finding an increase in the prevalence of EDS from 5.6% at baseline to 40.8% after 8 years, with EDS becoming non-resistible and more persistent over time (Gjerstad et al., 2006).

Longstanding evidence shows that therapy with levodopa alone or levodopa associated with dopamine agonists increases the risk of EDS in PD patients, linking higher LEDD with more severe EDS (Feng et al., 2021). Patients treated with pramipexole were found to have more EDS than patients treated with levodopa (Holloway et al., 2004); patients on a levodopa monotherapy experienced a significantly lower risk of sleep attacks than patients on dopamine agonists alone or on combination therapy (Paus et al., 2003). Interestingly, a 5-year follow-up study with PD patients with *de novo* diagnosis who underwent therapy (Tholfsen et al., 2015) has found that the main risk factor for developing EDS was a high ESS score (specifically between 6 and 10) at baseline, suggesting that dopaminergic drugs may increase daytime sleepiness, especially in patients with a higher propensity to somnolence before the beginning of the treatment. The disease severity of PD has been widely related to EDS since in early stages, where higher scores in the activities of daily living (ADLs) section of the Movement Disorder Society Unified Parkinson's Disease Rating Scale (MDS-UPDRS) have been related to more EDS (Tholfsen et al., 2015). Moreover, a recent meta-analysis showed that the scores of the MDS-UPDRS part III and Hoehn and Yahr (HY) stages were higher in PD patients with EDS (respectively, 3.02 and 0.23 points higher; Feng et al., 2021). These data were confirmed by a recent longitudinal study on 423 PD patients, showing that higher MDS-UPDRS part III scores during follow-up were related to higher ESS scores (Liu et al., 2023).

Finally, non-motor symptoms, such as cognitive impairment, autonomic dysfunction, or psychiatric disorders, are associated with EDS in PD patients. Cognitive impairment can occur in all stages of PD, with a wide spectrum from mild cognitive impairment (MCI) to dementia (Parkinson's Disease Dementia - PDD; Aarsland et al., 2021), patients with EDS were found to have additional features of cognitive impairment (global cognitive issues, executive dysfunction, and worse processing speed) than patients without EDS (Jester et al., 2020). The issue of EDS and its relationship with cognitive impairment may be relevant during the clinical interview: for this reason, the presence of caregivers is crucial when assessing EDS in these patients. Furthermore, deteriorated autonomic function assessed with the Scale for Outcomes in Parkinson's disease – Autonomic Symptoms (SCOPA-AUT) was related to EDS in PD patients and could worsen the ADLs in these patients (Huang et al., 2022). Orthostatic hypotension may be particularly invalidating, and its presence should be assessed by clinicians when patients report fatigability and difficulty in activities worsening with orthostatism. Anxiety and depression, examined by subjective questionnaires, were found to have a weak but significant correlation with EDS in PD patients, although there is still little evidence in the literature (Wen et al., 2017). No association was found with apathetic symptoms (Maggi et al., 2023), but it is important to differentiate them from excessive daytime sleepiness during the clinical interview.

## Pathophysiology

The etiology of EDS in PD is multifactorial (Happe, 2003), being either secondary, for example, to anti-parkinsonian drugs or to the presence of other sleep disorders and circadian rhythm dysfunction, as well as an integral part of the pathophysiological mechanisms underlying PD (primary EDS in PD).

### Primary EDS in PD patients

**Figure 1:**
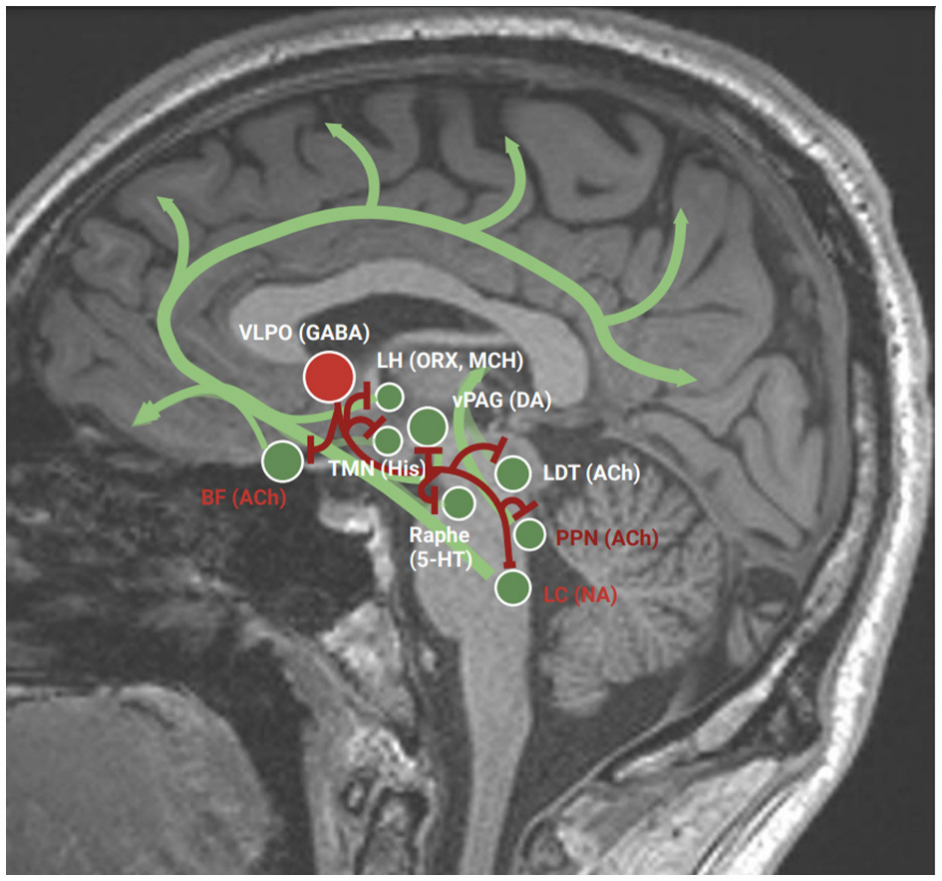
Wake-promoting and sleep-promoting nuclei. Green dots, wake-promoting nuclei; green line, main connection of wake-promoting nuclei; red dot, sleep-promoting nucleus; red line, main connection of sleep-promoting nuclei; red writings, impaired nuclei in Parkinson's disease; ACh, acetylcholine; BF, basal forebrain; DA, dopamine; GABA, gamma-aminobutyric acid; His, histamine; LC, locus coeruleus; MCH, melanin-concentrating hormone; ORX, orexin; PPN, pedunculopontine tegmental nucleus; TMN, tuberomammillary nucleus; vPAG, ventral periaqueductal gray; 5-HT, 5-hydroxytryptamin (serotonin).

There is growing evidence in the literature that the spreading of neurodegeneration related to PD pathophysiology may involve regions regulating the sleep–wake transition and maintenance (Figure 1). Patients with EDS presented less overnight decline of slow-wave activity (probably highlighting an incomplete sleep pressure dissipation through the night) and less whole-night spindle frequency activity (probably related to an altered thalamic activity during sleep) regardless of patients' clinical characteristics in a recent study by Schreiner et al. (2023). Moreover, some postmortem studies showed a cellular loss in PD patients for two wake-promoting nuclei: the pedunculopontine tegmental nucleus (cholinergic) and the locus coeruleus (noradrenergic; Giguère et al., 2018). Furthermore, using magnetic resonance imaging (MRI), several studies have attempted to correlate changes in brain structures with EDS in the PD population. One study has related fornix degeneration to EDS in PD patients (Matsui et al., 2006), while another one found distinct gray matter atrophy in the frontal, temporal, and occipital lobes and the nucleus basalis of Meynert in PD patients with EDS, compared with those without EDS and controls (Kato et al., 2012). Using functional MRI, it has been observed that PD patients with EDS have an increase of spontaneous neural activity in the left paracentral lobule while reduced activity in the left cerebellum and inferior frontal gyrus (Wen et al., 2016). Using single-photon emission computed tomography [in particular (123I) FP-CIT SPECT], two studies in early PD patients have found a correlation between DA transporter loss in the striatum, the caudate nucleus, and the putamen and higher ESS scores, suggesting that dopaminergic nigrostriatal degeneration at this stage might play a role in the development of EDS (Happe et al., 2007; Yousaf et al., 2018). The impairment of other wake-promoting nuclei, such as raphe serotoninergic neurons or ventral periaqueductal gray matter, has been studied in PD patients but still without sufficient evidence (Giguère et al., 2018). An impairment of the orexinergic system might play a role in the pathophysiology of EDS in PD patients. Orexin-releasing neurons have connections with both sleep-promoting nuclei and wake-promoting nuclei, resulting in promoting wakefulness and the stability of sleep–wake system (Zeitzer et al., 2003; Schwartz and Roth, 2008). There is some evidence of orexinergic neuronal loss in PD patients (Thannickal et al., 2007), together with lower CSF levels of orexin-A (Kumar et al., 2021). Moreover, a recent cross-sectional study investigating the role of narcolepsy human leukocyte antigen (HLA)–risk allele DQB1^*^0602 in PD patients found that HLA-positive PD patients were three times more likely to develop EDS than HLA-negative PD patients (Adam et al., 2022). The authors have not found differences in nighttime sleep between the two groups, suggesting that this phenotype could be a cause of EDS independent of nocturnal sleep disturbances.

Circadian rhythmicity is regulated by external factors (especially light as well as temperature and physical activity) and endogenous factors (age and hormones, such as melatonin; Patke et al., 2020). Melatonin secretion has been found to be more significantly decreased in PD patients with EDS compared to those without EDS (Videnovic et al., 2014). Light therapy and exercise therapy (used to restore circadian rhythmicity) can improve EDS in PD patients (Videnovic et al., 2017), suggesting a role of circadian rhythm dysfunction in the pathogenesis of EDS in PD patients, although there is a need for larger studies to confirm these results.

### Secondary EDS in PD patients

Other sleep disorders are common in PD patients and frequently coexist with EDS. The relationship between obstructive sleep apnea (OSA) and EDS in PD patients is still not well defined. Several studies revealed no difference in the incidence of OSA between PD patients with EDS and those without EDS (Crosta et al., 2017); others found that ESS scores were higher in PD patients with OSA than those without OSA and MSLT <8 min was related to higher apnea-hypopnea index (AHI) in PD patients (Lelieveld et al., 2012). OSA-related sleep fragmentation may produce EDS due to a lack of restorative nighttime sleep, and interestingly, intermittent hypoxia might produce neuroinflammation and the consequential neuronal damage in some wake-promoting areas could lead to the development of EDS in PD patients (Lal et al., 2021). PD patients may present sleep-related movements such as periodic limb movements (PLM), but their relationship with sleep disruption and daytime sleepiness is still not clear. Although restless legs syndrome (RLS) may be associated with PD, clinicians must be aware that, while it may be a cause of insomnia, it is not related to daytime sleepiness. Some studies showed that PD patients with rapid eye movement (REM) sleep behavior disorder (RBD) had more EDS compared to those without RBD (Rolinski et al., 2014; Dauvilliers et al., 2018). This is not likely related to sleep disruption but may be interestingly related to the simultaneous degeneration of the brainstem areas that regulate REM atonia (the subcoeruleus complex) and awakening (Kotagal et al., 2012; Iranzo et al., 2013; the pedunculopontine nucleus), supporting the hypothesis of a primary sleepiness in PD patients.

Antiparkinsonian dopaminergic medications, such as levodopa and dopamine (DA) agonists, are extensively accepted causes of EDS due to their sedative side effects in PD patients (Arnulf and Leu-Semenescu, 2009). Levodopa, the precursor of DA, has been reported to increase EDS as measured with both subjective (ESS) and objective (MSLT) methods (Arnulf et al., 2002). Among DA agonists, patients treated with pramipexole have shown a 4.98-fold risk factor of developing EDS compared to controls and a 2.2-fold risk factor compared to patients treated with levodopa (Etminan et al., 2001; Avorn et al., 2005). Furthermore, combination therapy with both levodopa and pramipexole has seen affecting EDS more than monotherapy (Paus et al., 2003). Similarly, treatment with rotigotine (Watts et al., 2007), apomorphine (Pahwa et al., 2007) and entacapone (Koller et al., 2005) have been related to increased EDS in PD patients. Several studies have found no relationship between the use of ropinirole and the occurrence of EDS (Chaudhuri et al., 2012), and one study has shown that ESS scores were significantly decreased in patients after switching to ropinirole prolonged-release from ropinirole immediate-release (Dusek et al., 2010). At the same time, there are some dopaminergic medications that appeared to improve EDS in PD patients. For example, piribedil (Eggert et al., 2014) and amantadine (Mehta et al., 2021) have been described as reducing EDS compared to placebo. In addition, some recent studies have shown that monoamine oxidase B (MAO-B) inhibitors (such as selegiline, rasagiline, and safinamide) have significantly reduced the ESS score in treated patients compared to placebo (Lyons et al., 2010; Hauser et al., 2014; Santos García et al., 2022). Overall, higher LEDD is related to EDS in PD patients, suggesting that the dose of dopaminergic agents is crucial. In healthy subjects, low doses of DA, levodopa, and DA agonists seem to promote sleep through D2-like inhibitory receptors, while high doses promoted alertness through D1-like and D2-like postsynaptic receptors (Valko et al., 2010). Moreover, selective D1 receptor agonist has been shown to reduce sleepiness, while most D2 receptor agonists could induce EDS in PD patients. It has been hypothesized that these different effects of dopaminergic drugs on EDS could be due to a different affinity for D1 and D2 receptors (Maggi et al., 2023).

## Diagnostic workup

The diagnostic workup of EDS in PD patients should focus on identifying the presence of excessive sleepiness and should differentiate primary from secondary EDS (examples from clinical practice are given in Boxes 1, 2). Even though there is no consensus on a diagnostic algorithm in these patients, we propose a multistep approach based on the current literature.

Box 1 **A case of primary EDS in PD: a work of exclusion.**

*Brief history*

A 66-year-old woman with an eight-year history of PD referred to our sleep center in June 2021 for the onset of excessive daytime sleepiness 2 years before (at the age of 64), referred as sudden not resistible sleep attacks during the day, generally three or four per week in relaxed situations, such as watching TV, being a passenger in a car (not the driver), or sitting on a chair listening to conferences, overall in relaxed situations. The patient had a previous non-significant medical history. She referred a fragmented nocturnal sleep, with 2–3 prolonged awakenings after sleep onset. Moreover, she usually had a 3-h nap after lunch. Her motor symptoms began at 58 years with bradykinesia at right limbs, with bilateralization at 62 y.o. and recent emergence of limb dyskinesias in ON state. She started therapy at 59 years with Levodopa/Carbidopa and after few months added Pramipexole. Her non-motor symptoms also included constipation.

*Our evaluation*

At hospital admission she had a UPDRS part III of 31 in OFF state, ESS was performed with a result of 10 points. EDS was not related to dopaminergic therapy changes, and from medical history and clinical interview no suspect of concurrent sleep disorder was brought out. To better characterize her sleepiness, a MSLT test was performed, showing a mean sleep latency of 4.5 minutes. After excluding possible confounding factors and secondary causes of EDS, a non-pharmacological strategy was preferred to a pharmacological therapy, in particular stressing the importance of sleep hygiene, with avoiding having naps during the day, and being exposed to bright light after waking up in the morning. After 6 months, at follow-up, due to a partial response to sleep hygiene interventions (sleep attacks were less frequent but still occurred once or twice per week), a vPSG was performed in order to exclude possible secondary factors, such as unreferred sleep disorders, occurred after the beginning of the therapy. The vPSG showed no concurrent sleep disorder and a melatonin-based therapy was started, with an improvement in sleepiness symptoms at 3 months follow-up.

*Discussion*

With this clinical vignette, we underlined the importance of excluding other possible causes of EDS. Fatigue, mood disturbances and orthostatic hypotension were ruled out after the clinical history collection and in clinic evaluation, same as for drug-related EDS (no correlation with dopaminergic therapy administration and no use of sleep medication). No suspects of concurrent sleep disorders (particularly SBD and circadian rhythm disorders) emerged after clinical interview. The borderline value of ESS (10 points), lead to perform an objective measurement of EDS and we decided to perform MSLT that resulted above the pathological threshold. First therapeutical approach may be non-pharmacological, but after first line in not completely effective, one can perform another type of objective measurement (i.e., vPSG) to search for new-onset sleep disorder. Finally, we decided to start a pharmacological therapy with a non-stimulant drug (melatonin), with improvements in symptoms.

Box 2 **Secondary EDS in PD: seeking the culprit.**

*Brief history*

A 65-year-old male with a five-year history of PD referred to our sleep center in June 2023 for investigating the possible causes of a worsening daytime sleepiness gradually appeared during the last year, referred as sudden sleep attacks during the day, especially in the morning, while reading or working at the computer. The patient had a previous non-significant medical history. His motor symptoms began at 59 years with resting tremor at right limbs, with bilateralization at 64 y.o. He started dopaminergic therapy with Pramipexole prolonged release at 8pm in 2018. In September 2022, he added Levodopa/Carbidopa in association with Pramipexole: he gradually started complaining sleepiness symptoms. For this reason, he decreased dopamine agonist dosage with partial benefit on sleepiness (reduction in sleep attack frequency), while reporting reappearance of tremor. His non-motor symptoms also included iposmia, constipation and urinary incontinence and he complained about a recent worsening in attention and memory performances.

*Our evaluation*

At hospital admission he had a BMI of 31.1 kg/m^2^ and UPDRS part III of 22 in OFF. ESS was performed with a result of 11 points. In the suspect of a drug-related secondary EDS, patient's sleepiness was monitored daily with diaries and ESS, finding no correlation between therapy administration and sleep attacks. Moreover, when questioned, the patient referred snoring and sleep apnoea symptoms, begun since the age of 50. Hence, a polygraphy was performed and showed a moderate OSA (overall AHI 20.7), greatly influenced by supine position (supine AHI 90). Based on these results, CPAP was titrated and prescribed at the most suitable pressure for the patient. At follow-up, after 1 month of therapy, an improvement of sleepiness symptoms was found, and dopaminergic therapy was increased due to the overall worsening of motor symptoms during last year; a further follow-up confirmed the improvement of daytime sleepiness and showed an improved in motor symptoms.

*Discussion*

With this clinical vignette, we exemplified the proposed diagnostic algorithm in clinical practice. Indeed, we started with a careful collection of patient's history (step 1), then we administered the ESS (step 2) and a diary to monitor sleepiness through the day and finally we performed an objective evaluation (step 3) to confirm the anamnestic suspect and started the specific therapy. From the beginning, we suspected a secondary cause of patient's daytime sleepiness: first, our suspect went to the association between Levodopa/Carbidopa and Pramipexole, but the careful monitoring of EDS through diaries excluded a correlation with sleep attacks. Moreover, the anamnestic detail of probable OSA lead us to perform a polygraphy with the evidence of severe positional OSA and to begin CPAP titration, with an improvement of sleepiness symptoms. Although DA therapy may have played a role in this patient's sleepiness, we could conclude that his EDS was secondary to a concurrent SBD.

### Step 1: clinical interview and physical examination

Considering both inpatients and outpatients, the first approach to excessive sleepiness begins with taking a detailed medical history. PD is a complex clinical condition, with different motor and non-motor issues and different levels of autonomy among patients. Before sleep-related information, a detailed PD history is important to have a general idea of the patient's risk of experiencing EDS not only at present but also in the future: disease duration and severity, medication history (including not only dopaminergic but any other drug), and presence of other non-motor symptoms (especially cognitive, autonomic) should be assessed. Then, the clinician should obtain sleep-related information, including bedtime (both during workdays and weekends/holidays), sleep latency, the number and duration of awakenings during nighttime, presence of snoring or apneas (and related symptoms such as awakenings with gasping for air or mouth dryness), and any limb movements or complex behaviors during sleep. Of course, during the clinical interview, especially when asking sleep-related questions, the presence of the patient's bedpartner is crucial, and any information taken without the bedpartner should be considered not completely reliable. As mentioned earlier, sometimes patients may not distinguish sleepiness symptoms from fatigue or muscular weakness, and for this reason, other and more specific questions should be asked to the patient rather than “Do you feel sleepy during daytime?” such as “*Do you take naps?*” or “*Do you fall asleep at times you do not want to?*” or “*Do you struggle to stay awake during the day?*” (Bodkin and Manchanda, 2011). The answers to these questions may differentiate sleepiness from a general lack of energy (including fatigue and fatigability) or motivation (including depression and/or apathy) that may be concurrent or not (Box 3). The neurological examination is important to explore extrapyramidal signs (such as bradykinesia, rigidity, tremor, and gait instability), and the presence of orthostatic hypotension should be assessed. Moreover, observing the presence of overweight and large neck circumference may increase the likelihood of concurrent sleep-disordered breathing (Bhat and Chokroverty, 2017). If there is a suspicion of cognitive impairment, the Mini-Mental Status Examination or the Montreal Cognitive Assessment scale, which takes a few minutes to be administered during the visit, may be useful to assess interview “reliability”: the presence of bedpartners or caregivers is necessary during the whole interview when the patient has cognitive impairment. However, it is important to underline that, even without cognitive impairment, the presence of the patient's bed partner is necessary when collecting sleep-related information.

Box 3 **Proposed semi-structured interview for collecting sleepiness-related history.**

1. Risk factors-related questions (to both patients and bedpartners):
• When has the patient firstly experienced symptoms of Parkinson's disease? When has the patient been diagnosed?
• Does the patient experience dizziness while in orthostatism? Has he/she ever experienced syncope? Do the patient present urinary incontinence or other conditions that may lead to multiple awakenins during night time sleep?
• Is the patient autonomous in all daily life activities or does he/she need help in any of them?
• Which therapy is the patient using for Parkinsonian symptoms? At what dose? Does the patient present any disturbance before or after taking the therapy? Does the patient use neuroleptics, benzodiazepines or antidepressants?
2. Sleep-related questions (to both patients and bedpartners):
• What time does the patient usually go to bed and get up? Are there any differences in weekends or holidays than in workdays?
• How long does the patient usually fall asleep?
• How often does the patient wake up during night time sleep? Are there any particular reasons?
• Does the patient snore or spend many seconds without breathing during sleep?
• Does the patient usually move the limbs during sleep? Is it possible to recognize an aggressive behavior? Does the patient talk during sleep? If there are any complex motor behaviors, in what part of the night do they occur (right after falling asleep, early in the morning)?
3. Daytime sleepiness-related questions (to both patients and caregivers):
• Does the patient take naps during the day? How long are them?
• Does the patient fall asleep at inappropriate situations during the day or at times the patient does not want to?
• Does the patient struggle to stay awake during the day?
4. Fatigue-related questions (to both patients and caregiver):
• Does the patient have a lack of energy while doing normal daily activities?
• Does the patient feel physically or mentally exhausted?
• Does the patient have a lack of motivation?

### Step 2: questionnaires

In addition to the clinical interview, the administration of specific questionnaires to both patients and bedpartners may help the clinician to better characterize excessive daytime sleepiness. PDSS (score >18 defines a clinically relevant PD-specific sleep disturbance) and SCOPA-SLEEP are questionnaires prepared for PD patients and should be preferred. ESS should be used to quantify EDS using a cutoff of >10 points and may be useful also for monitoring sleepiness after therapeutic intervention and during the follow-up. Moreover, the Pittsburgh Sleep Quality Index (Buysse et al., 1989) may be used to investigate sleep quality (score >5 indicates poor sleep quality) and may be administered again during follow-up. There are also several different scales to assess the presence of fatigue if suspected: the most used is the Fatigue Severity Scale (Herlofson and Larsen, 2002), with a clinically relevant cutoff of >36. If concurrent sleep disorders are suspected during the clinical interview, specific questionnaires could be administered depending on the disorder, for example, the STOP-Bang (Nagappa et al., 2015) questionnaire if OSA is suspected. At the end of the clinical interview, the physical examination, and the administration of questionnaires, clinicians should assess the presence of EDS or not and suspect that the presence of EDS is secondary to another condition or not. If EDS is likely to be related to PD medications or other drugs, a withdrawal or reduction of the therapy is recommended before performing any objective assessment (see “Step 3: objective assessment” section).

### Step 3: objective assessment

**Figure 2:**
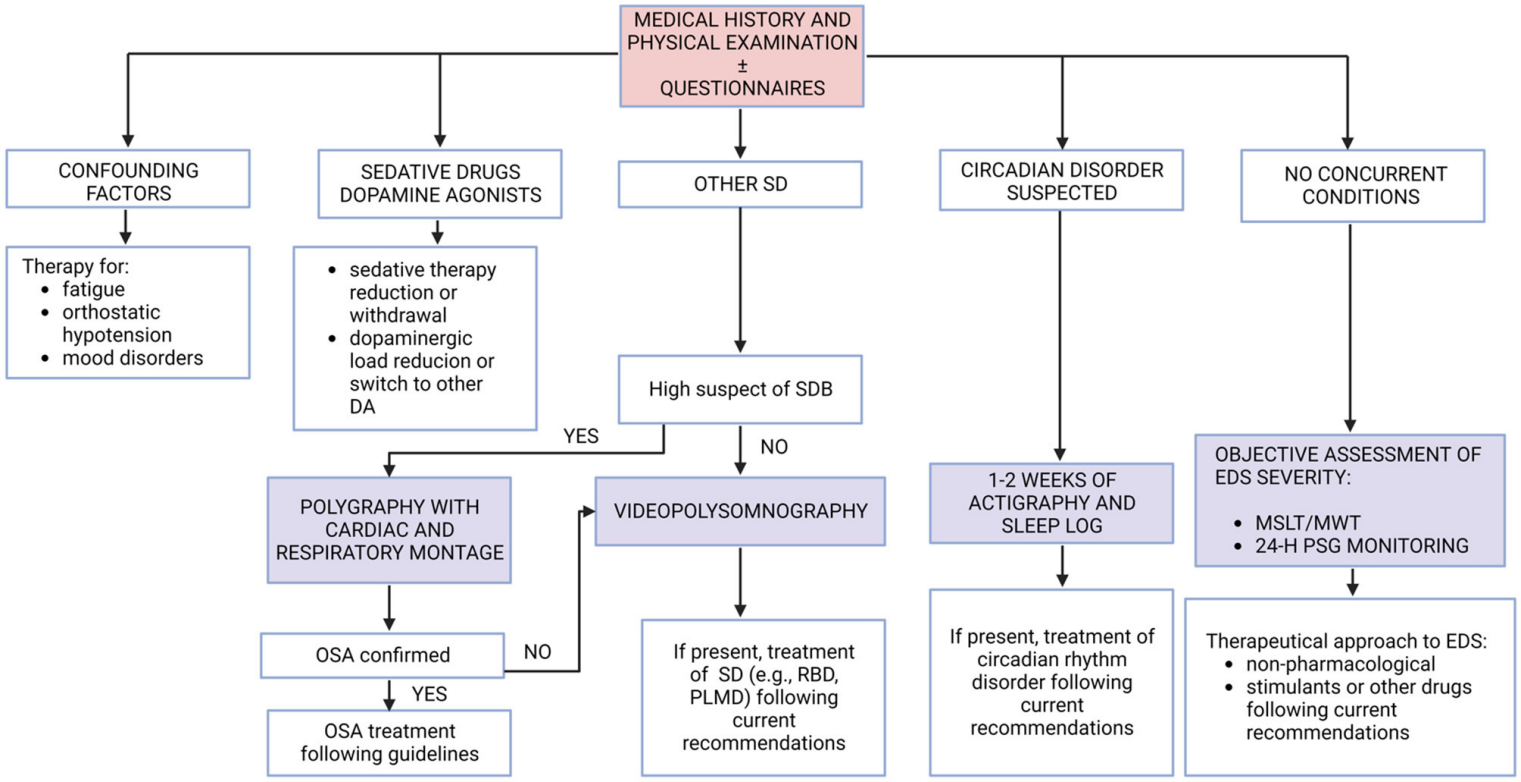
Summary of diagnostic steps to assess EDS in PD patients. DA, dopamine agonists; EDS, excessive daytime sleepiness; MSLT, multiple sleep latency test; MWT, maintenance of wakefulness test; OSA, obstructive sleep apnea; PD, Parkinson's disease; PLMD, periodic limb movement disorder; PSG, polysomnography; RBD, rapid eye movement sleep behavior disorder; SD, sleep disorder.

After the causative role of medication is excluded and any possible mimics (such as fatigue) are excluded by the detailed diagnostic workup of Step 1 and Step 2, the last and most precise way to assess EDS is by objective methods. As mentioned earlier, an objective EDS assessment has shown a higher prevalence of daytime sleepiness in PD patients rather than EDS assessments based on questionnaires probably due to an underestimation of the symptoms by the patients. There are many different objective methods to assess EDS and to investigate its possible causes (Figure 2). A 7–14-day actigraphic monitoring, together with a well-compiled sleep log, may be useful for detecting circadian dysfunction and monitoring the efficacy of the specific treatment (see “Current therapeutic approaches” section). For example, when patients report later sleep onset and waking time, with more restorative sleep during weekends and holidays than in workdays, circadian rhythm dysfunction (i.e., delayed sleep-wake phase disorder) should be suspected. If sleep-disordered breathing (SDB) is suspected by clinical interview (and by physical examination), a polygraphy test with cardiac and respiratory montage following the current guidelines is mandatory, and clinicians should start the specific treatment for SDB before treating EDS. If, during the collection of sleep-related history of the patient (or more frequently the bed partner), there complains about limb movements or complex behaviors during sleep time, performing a video-polysomnography is mandatory following the current guidelines to detect an underlying sleep disorder (such as PLM disorder or RBD), and clinicians should start specific treatment for the disorder. Considering daytime sleepiness objective assessment and its severity, performing MLST and/or MWT may be useful especially when clinical history and questionnaire results are not concordant to investigate the severity of EDS before starting a therapy. However, the evidence about MSLT and MWT reliability in PD patients is not as clear as in other populations, where they are well studied and extensively recommended (MSLT is part of the diagnostic criteria in narcolepsy). Finally, a 24-h polysomnographic monitoring should be considered when clinicians want to study both nocturnal and diurnal sleep and its characteristics, including nocturnal sleep fragmentation and daytime naps. Moreover, it is easier to perform polysomnographic monitoring on inpatients, but there are also tools for home monitoring that could be used in outpatients. Objective measures may also be performed or repeated over time, especially when the first therapeutic approach is or becomes ineffective. Indeed, underlying sleep disorders may not be revealed at the first objective assessment or may be developed during follow-up time.

## Current therapeutic approaches

There is a lack of specific guidelines for a standardized treatment of EDS in PD patients, probably due to the heterogeneity of causes of EDS and the different clinical pictures among PD patients.

As a general advice, treatment should be individualized to the patient and directed at the underlying causes when present. However, as mentioned before, EDS should be considered as both part of the physiopathology of PD (due to the degeneration of specific nuclei) and should coexist with other sleep disorders, complicating the therapeutic approach.

In 2010, the European Federation of Neurological Societies and Movement Disorder Society–European Section (Ferreira et al., 2013) outlined several recommendations for the management of EDS in PD, including assessing nocturnal sleep disturbances, improving nocturnal sleep (by reducing akinesia, tremor, and urinary frequency), recommending the person to stop driving, reducing/discontinuing sedative drugs, decreasing dopaminergic drugs (mainly DA agonists) or switching to other DA agonists, and finally adding modafinil or other wake-promoting agents.

As recommended, the treatment of EDS should include treatment of nighttime motor (akinesia and tremor) or non-motor (urinary) complications: for example, nighttime akinesia could be improved by introducing extended-release formulations of dopaminergic agents. Regarding DA agonist–related sleepiness, dosage reduction, monotherapy, or replacement with other dopaminergic medication (such as MAO-B inhibitors) with less risk of developing EDS could be considered in these patients. If patients are currently on therapy with antidepressants, neuroleptics, or benzodiazepines, drug withdrawal or reduction may be recommended (Loddo et al., 2017). If a primary sleep disorder is suspected by clinical evaluation, or confirmed by a PSG exam, it is important to focus on the management of the disorder before starting a specific therapy for EDS. For instance, patients with OSA treated with continuous positive airway pressure may improve EDS through subjective and objective improvement of sleep quality (Neikrug et al., 2014).

**Figure 3:**
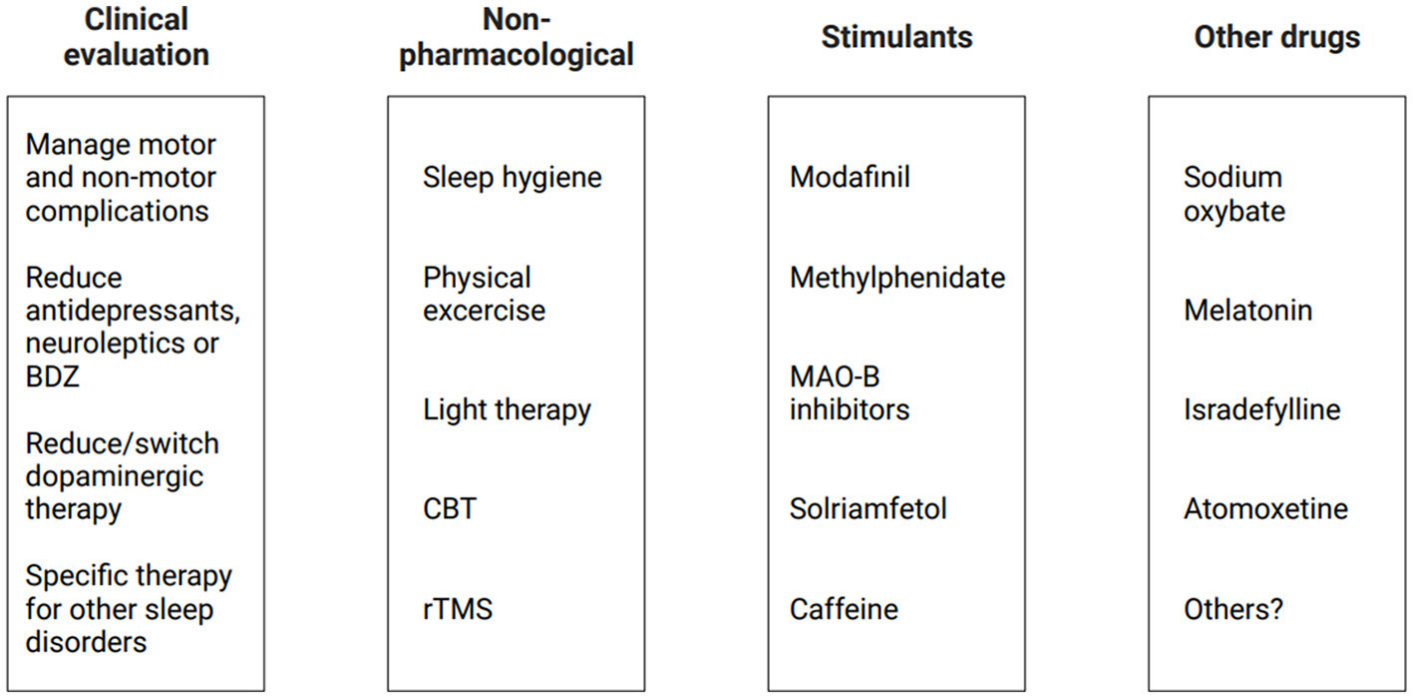
Current therapeutic approaches for EDS in PD patients. BDZ, benzodiazepines; CBT, cognitive behavioral therapy; EDS, excessive daytime sleepiness; MAO-B, monoamine oxidase B inhibitors; PD, Parkinson's disease; rTMS, repetitive transcranial magnetic stimulation.

If EDS persists even with adequate dopaminergic therapy adjustment and good management of coexisting sleep disorders, non-pharmacological strategies and other pharmacological approaches should be considered (Figure 3). However, as little evidence supports the efficacy and safety of pharmacological intervention in PD patients with EDS, it may be suggested to begin with a non-pharmacological approach.

### Non-pharmacological intervention

First, general sleep hygiene recommendations should be given to the patients, including regular bedtime and, if necessary, structured napping periods. Exercising during the day, including regular physical activity in the morning and scheduled rehabilitation, may improve many non-motor symptoms in PD, including a reduction of fatigue, an increase in sleep quality, and a reduction of EDS. In addition, outdoor activities and avoiding vigorous physical activity 3–4 h before sleeping might also be helpful in this process (van der Kolk and King, 2013; Schütz et al., 2022). When a circadian rhythm dysfunction is detected (e.g., delayed sleep–wake phase disorder), supplementary exposure to bright light using light therapy in the morning might have beneficial effects on EDS by influencing circadian rhythm; however, there is no consensus on a therapeutic protocol in PD patients, and the efficacy must be proved by more studies (Lin et al., 2021). Together with sleep hygiene recommendations, a psychological approach such as cognitive behavioral therapy (CBT) may be useful for changing dysfunctional behaviors; however, although there are some studies evaluating insomnia and CBT in PD patients, there are limited studies for its efficacy in EDS (Rios Romenets et al., 2013). Finally, an interesting non-pharmacological perspective for treating EDS could be repetitive transcranial magnetic stimulation (rTMS): one recent study used low frequency (<1 Hz) rTMS on the right dorsolateral prefrontal cortex in 25 PD patients, with an improvement in ESS scores (Zhang et al., 2022b). Further studies are needed to confirm these findings, but there is a growing interest in literature on the topic.

### Pharmacological strategies

A pharmacological approach may be considered if secondary causes of EDS are excluded or treated and non-pharmacological strategies fail to improve EDS, but there are still few clinical trials about wakefulness-promoting agents or stimulants, and they are conducted with a short-term follow-up.

A meta-analysis showed that modafinil (100–400 mg/day in clinical practice) reduced subjective EDS symptoms in PD patients measured with ESS scores (Rodrigues et al., 2016); however, other studies showed no difference when EDS was objectively measured (Knie et al., 2011). THN102 is a novel combination drug of modafinil and low-dose flecainide, and one study showed that it improved ESS scores in PD patients with EDS with good tolerability (Corvol et al., 2022). Cardiovascular side effects (increasing blood pressure and heart rate) should be considered in older adults and cardiopathic patients, and moreover, this drug might worsen insomnia symptoms (Sheng et al., 2013). Although it is approved for the treatment of EDS in narcolepsy, its use in PD patients has many limitations. An open-label study showed that methylphenidate (10–80 mg/d in clinical practice) reduced EDS in PD patients together with an improvement of motor and gait symptoms in combination or without levodopa (Devos et al., 2007). However, another study showed that there were described possible side effects such as insomnia, reduced appetite, and psychosis (Leonard et al., 2004). Further evidence is lacking regarding the efficacy and safety of this drug in PD patients. Recently, MAO-B inhibitors were used in trials on PD patients with EDS: an 8-week study showed selegiline improved ESS scores together with sleep quality and nocturnal motor symptoms (Zhang et al., 2022a); moreover, a 6-month study showed daytime sleepiness and quality of life improved in PD patients with the use of safinamide (Santos García et al., 2022). There is still little evidence about these drugs, and further studies are needed to assess their possible benefits. One recent study showed that solriamfetol could be an interesting alternative as a pharmacological approach to EDS in PD patients; in fact, it has been shown to reduce both subjective and objective EDS, although its real efficacy was not clear due to several limitations owing to its use in possible combinations with other dopaminergic drugs (Videnovic et al., 2021). More clinical trials are needed before recommending this drug as a therapeutic approach for EDS in PD patients. The use of caffeine has been under investigation for its potential neuroprotective effects, and it seems to mildly reduce the risk of developing PD (Noyce et al., 2012). Recent studies have shown different results when used as therapy (200–400 mg/d) for EDS in PD patients, with one study showing no differences in ESS scores (Postuma et al., 2012) and another one showing an improvement in EDS over the first 6 months of therapy (Postuma et al., 2017). The long-term effects of caffeine on EDS are still unclear, and further studies are needed.

Apart from wake-promoting agents, other pharmacological approaches were attempted in several studies. For example, interestingly, sodium oxybate (3–9 g/day) has been recently tested in PD patients with EDS showing an improvement of sleepiness and sleep quality with both subjective (ESS score) and objective (mean sleep latency, slow wave activity) parameters (Ondo et al., 2008; Büchele et al., 2018). This drug has multiple side effects, such as psychiatric complications, sleep apnea, and parasomnia. Although it might be efficacious for treating EDS in PD patients, its side effects could be dangerous, requiring extreme caution for its usage. Melatonin, alone or in combination with light therapy, may be useful when a concurrent circadian rhythm disorder (e.g., delayed sleep–wake phase disorder) is suspected, and it is also effective if the patients have concurrent RBD. Other therapeutic strategies include istradefylline (20–40 mg/day), which is approved by the United States Food and Drug Administration (FDA) as an add-on therapy for dyskinesia in PD and was shown to reduce EDS in PD in a 3-month study (Suzuki et al., 2017), but further studies are needed to prove its long-term efficacy and safety. One trial with atomoxetine (used as therapy for attention-deficit/hyperactivity disorder) showed an improvement in EDS and cognitive performance in PD patients, but there is a lack of other evidence and thus further studies are needed (Weintraub et al., 2010). Although there are no studies in the literature on the use of a histamine type 3 receptor antagonist, pitolisant, in PD patients with EDS, the growing evidence of its efficacy in other types of hypersomnolence may be a hint for future trials on these patients.

In conclusion, there is no recommendation for specific drugs for long-term therapies in these patients (Seppi et al., 2011). In 2021, the American Academy of Sleep Medicine (AASM) clinical practice guideline for treating hypersomnolence (Maski et al., 2021) suggested using modafinil or sodium oxybate in PD patients, with no other drug having enough evidence in the literature.

## Conclusion

Excessive daytime sleepiness is a common non-motor symptom in PD. In this narrative review, we suggest a diagnostic approach to EDS that can be replicated by clinicians in both inpatients and outpatients with PD. First, confounding factors and concurrent medical conditions should be excluded. Based on a semistructured interview, collecting the sleep-related medical history is the cornerstone of this diagnostic workup and specific questionnaires may help clinicians to address further diagnostic steps; however, in our opinion, objective measurements of daytime sleepiness remain essential for a correct clinical evaluation and therapeutical management. The growing interest in the relationship between daytime sleepiness and neurodegeneration of specific brain regions in PD may lead the scientific community to developing new therapies for this clinical condition, but there is still a lack of evidence on this topic, and further studies are needed. Apart from the dopaminergic system, other pathways may be related to daytime sleepiness (e.g., histaminergic or orexinergic systems), which is possibly more specific for this symptom than other non-motor manifestations of PD: it may be interesting to characterize these pathways in PD patients with EDS. At the time of this review, there are several recommendations for the therapeutical management of these patients but no specific guidelines: more clinical trials are needed to test the efficacy and possible side effects of stimulants targeting different neurotransmitter pathways.

## Author contributions

FD: Writing—original draft. LB: Conceptualization, Methodology, Supervision, Writing—review & editing. GM: Writing—review & editing. GL: Writing—review & editing. AM: Writing—review & editing. CP: Writing—review & editing. MS: Writing—review & editing. FM: Writing—review & editing. FP: Conceptualization, Methodology, Supervision, Validation, Writing—review & editing.
